# Supplementary material for: Predictors of time to death among preterm neonates admitted to neonatal intensive care units at public hospitals in southern Ethiopia: A cohort study
Source: PLoS One. 2023 Oct 12;18(10):e0283143. doi: 10.1371/journal.pone.0283143 (PMC10569587; doi:10.1371/journal.pone.0283143)
Supplement: Attachment — Submitted filename: Manuscript review.docx [file pone.0283143.s002.docx]

Manuscript review:

Title: "Predictors of time to death among preterm neonates admitted to neonatal intensive care units at public hospitals in southern Ethiopia: A cohort study."

Summary comments: Generally well written manuscript. The subject of the paper, neonatal mortality is an important subject of public health importance.

Few comments below mainly related to typos should be addressed by the authors

**Introduction**

Line 46: Authors wrote “Preterm birth, defined as a live birth before 37 completed weeks of pregnancy(1) and remains one of the common causes of hospitalization……..”

They may either capture the sentence as “Preterm birth, defined as a live birth before 37 completed weeks of pregnancy(1) remains one of the common causes of hospitalization……..

”

Or “Preterm birth is defined as a live birth before 37 completed weeks of pregnancy(1) and remains one of the common causes of hospitalization ………

”

Line 49-51. The authors should review the tenses used. For instance, they state “Globally, the sub-Saharan Africa had the highest newborn mortality rate 27 deaths per 1,000 live births, followed by Central and Southern Asia (25 deaths per 1,000 live births) (3).

”

They should clarify whether they mean, Globally, sub-saharan Africa has… or they meant ‘’had’’ in which case it will be good to know what the current NMR is sub-Saharan Africa is.

Line 56-57: The sentence below is incomplete, please rephrase into a complete sentence: “Despite the national goal of reducing the NMR from 57 28 to 11 per 1000 by 2020 as part of the national newborn and child survival strategy (2015-2020) 58 (6).”

Line 54-58. Question: Why do we have differences in the national Health Sector Transformation Plan (HSTP-I) target of reducing NMR to 10 deaths per 1,000 live births by 2020 and the national goal of reducing the NMR from 28 to 11 per 1000 by 2020. Are they different agencies?

Line 62: “Ethiopia's neonatal mortality rate unacceptably high”

It should rather read “Ethiopia's neonatal mortality rate is unacceptably high”

**Methods**

Lines 76 and 80, please remove duplication of sentence as captured below:

Line 76: neonatology ward accommodate 16beds in including 8 total beds from NICU ward.

Line 80: there is 16 beds in neonatology ward including 8 total beds from NICU ward.

Comment: Repetition of sentence

Line 97 to 99:

“The checklist for this study was adapted from the similar literatures, neonates’ medical card and

99 logbook and modified after reviewing of other related literatures.”

This may read better as “The checklist for this study was adapted from neonates’ medical card and logbook with modifications based on literature review.” Avoid use of the term “literatures”. Literature can be used as plural.

Line 99: “A checklist consisted of the…”

Please use “The checklist consisted of the…..”

Line 97 to 104: In summary, the section on data collection can be better organized.

See suggestion below based on what the authors been wrote:

“The data were extracted from each individual neonate’s medical card and logbook using a structured checklist. The checklist consisted of information on maternal and neonatal socio demographic factors, neonatal related factors, maternal medical and obstetric-related factors.

Since the data was collected from neonates’ medical card, the data collectors were trained on how to extract appropriate data and there was close daily monitoring and supervision at the data collection site.”

**Results**: Line 153: “Four hundred and five neonates (79%) were cried immediately”

Comment; Please remove “were”

**Discussion:**

Line 276-277: “This study revealed that 9-fold increase in hazard to mortality among preterm neonates who had not received KMC compared to those who were received KMC during hospital stay.”

Rephrase: “This study revealed 9-fold increase in hazard to mortality among preterm neonates who had not received KMC compared to those who received KMC during hospital stay.”

The manuscript will benefit from a conclusion paragraph that summarizes the findings and way forward. The conclusion at the abstract section is appropriate and can be used or adapted for this section.

See conclusion of abstract below: “Conclusion: Despite admission to NICU for advanced care and follow up, mortality of preterm neonates was found to be high in the study settings. Addressing major intrapartum complications is required to improve survival of neonates admitted to NICU.”
